# The association between airflow limitation and blood eosinophil levels with treatment outcomes in patients with chronic obstructive pulmonary disease and prolonged mechanical ventilation

**DOI:** 10.1038/s41598-019-49918-z

**Published:** 2019-09-17

**Authors:** Wei-Chang Huang, Chen-Cheng Huang, Pi-Chu Wu, Chao-Jung Chen, Ya-Hua Cheng, Hui-Chen Chen, Ching-Hsiao Lee, Ming-Feng Wu, Jeng-Yuan Hsu

**Affiliations:** 1 Division of Chest Medicine, Department of Internal Medicine, Taichung Veterans General Hospital, Taichung, 407 Taiwan; 2 Department of Medical Technology, Jen-Teh Junior College of Medicine, Nursing and Management, Miaoli, 356 Taiwan; 3 Department of Life Sciences, National Chung Hsing University, Taichung, 402 Taiwan; 4 Department of Industrial Engineering and Enterprise Information, Tunghai University, Taichung, 407 Taiwan; 5 Division of Chest Medicine, Department of Internal Medicine, Taichung Hospital, Ministry of Health and Welfare, Taichung, 403 Taiwan; 6 Nursing Department, Taichung Veterans General Hospital, Taichung, 407 Taiwan; 7 Division of Critical Care and Respiratory Therapy, Department of Internal Medicine, Taichung Veterans General Hospital, Taichung, 407 Taiwan; 8 Department of Medical Laboratory Science and Biotechnology, Central Taiwan University of Science and Technology, Taichung, 406 Taiwan; 9 Division of Clinical Research, Department of Medical Research, Taichung Veterans General Hospital, Taichung, 407 Taiwan; 10 School of Medicine, China Medical University, Taichung, 404 Taiwan; 11 School of Physical Therapy, Chung-Shan Medical University, Taichung, 402 Taiwan

**Keywords:** Outcomes research, Risk factors

## Abstract

The clinical implications of airflow limitation severity and blood eosinophil level in patients with chronic obstructive pulmonary disease (COPD) and prolonged mechanical ventilation (PMV) are unknown. Thus, this study aimed to identify whether or not these two indicators were significantly associated with short-term in-respiratory care center (RCC) treatment outcomes in this population. Of all participants (n = 181) in this retrospective cross-sectional study, 41.4%, 40.9%, 8.3%, and 52.5% had prolonged RCC admission (RCC length of stay >21 days), failed weaning, death, and any adverse outcomes of interest, respectively. Compared to participants without any adverse outcomes of interest, moderate (the Global Initiative for Chronic Obstructive Lung Disease (GOLD) II) and/or severe (GOLD III) airflow limitation were significantly associated with short-term in-RCC adverse outcomes in terms of failed weaning (for III versus I, OR = 15.06, p = 0.003) and having any adverse outcomes of interest (for II versus I, OR = 17.66, p = 0.002; for III versus I, OR = 37.07, p = 0.000) though the severity of airflow limitation did not have associations with prolonged RCC admission and death after adjustment. Meanwhile, blood eosinophilia defined by various cut-off values was not associated with any adverse outcomes. The findings have significant clinical implications and are useful in the management of patients with COPD and PMV.

## Introduction

The number of patients requiring prolonged mechanical ventilation (PMV), generally defined as at least 14–21 days of continuous mechanical ventilation, is rapidly increasing worldwide due to an aging population, a greater number of co-morbidities and advances in critical care^1–5^, leading to increased medical resource utilization and financial burden. Chronic obstructive pulmonary disease (COPD), characterized by persistent respiratory symptoms and airflow limitation that is usually caused by significant exposure to noxious gases or particles, is present in 8.6% of critically ill patients and in 46% to 59% of patients with PMV^6–9^. Moreover, the presence of COPD, either as the cause of admission to an intensive care unit (ICU) or as a co-morbidity, is associated with PMV and has been shown to be an independent risk factor for mortality in both critically ill patients and patients with PMV^7,10^.

To the best of our knowledge, only one previous study has explored factors associated with treatment outcomes in patients with COPD and PMV, which found that better long-term survival was associated with younger age, shorter length of stay in the ICU and the respiratory care center (RCC), and provision of maintenance non-invasive positive pressure ventilation after weaning^11^. Thus, little is known regarding the factors associated with various treatment outcomes in this population.

Previously, forced expiratory volume in one second (FEV1) % predicted, rather than blood eosinophil levels, is an objective measure of airflow limitation severity used in clinical practice and in therapeutic trials in patients with COPD. Emerging evidence indicates that FEV1 by itself is a poor predictor of treatment outcomes in terms of the risk of exacerbations and mortality in patients with stable COPD^6,12,13^. In contrast, it has been shown that blood eosinophilia, defined as either >2% or >300 cells/μL, is associated with a higher risk of exacerbations in patients with stable COPD^14^. This transition relocates these two indicators in the management of COPD. Nevertheless, whether or not the severity of airflow limitation and blood eosinophil level are significant risk factors for treatment outcomes in patients with COPD and PMV remains unknown.

We hypothesized that airflow limitation severity and blood eosinophil level may be significant factors associated with treatment outcomes in patients with COPD and PMV. Therefore, the aims of this study were to identify whether the severity of airflow limitation as determined by FEV1% predicted and blood eosinophil level were independent risk factors associated with short-term treatment outcomes including the length of stay, weaning outcomes, and mortality, in this population.

## Results

### Baseline demographics and characteristics of the enrolled participants

**Figure 1:**
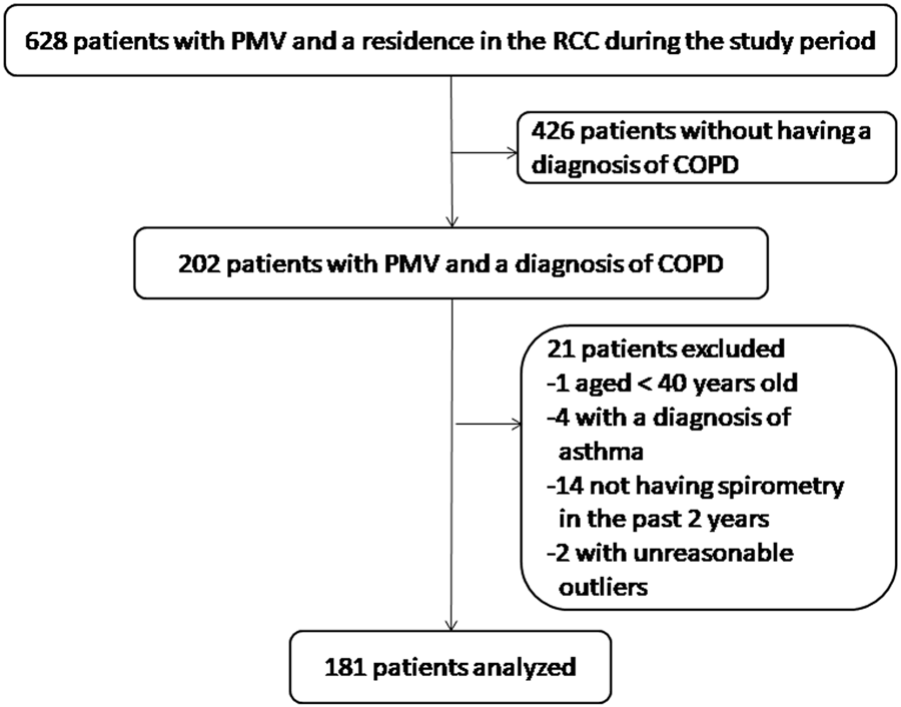
The patient enrollment flow chart. Abbreviations: COPD, chronic obstructive pulmonary disease; PMV, prolonged mechanical ventilation; RCC, respiratory care center.

Figure 1 presents the patient enrollment flow chart. A total of 181 patients were analyzed. Table 1 provides a selected subset of the baseline information of the enrolled participants. The entire list of the baseline demographics and characteristics of the enrolled patients can be found as Supplementary Table S1. The mean age of the participants was 75.4 ± 12.3 years, and the majority were male and had a history of smoking. 87.3% (158/181) of the participants had moderate-to-severe airflow limitation. All of the participants received short-acting bronchodilators as the pharmacological therapy for COPD in the RCC.

**Table 1:** Demographic and clinical characteristics of all participants and study groups categorized by respiratory care center length of stay, weaning outcomes, and mortality.

	Individuals with RCC length of stay >21 days (n = 75)	Individuals with failed weaning (n = 74)	Individuals with death (n = 15)	Individuals with any adverse outcomes (n = 95)	Individuals without any adverse outcomes (n = 86)	Total (n = 181)
Age (years)	78.0 ± 10.4	77.7 ± 11.3	76.4 ± 14.0	77.1 ± 11.2	73.5 ± 13.1	75.4 ± 12.3
Male gender	65 (86.7%)	67 (90.5%)	14 (93.3%)	84 (88.4%)	71 (82.6%)	155 (85.6%)
Smoking history						
Blood absoluteNever	7 (9.3%)	9 (12.2%)	1 (6.7%)	11 (11.6%)	17 (19.8%)	28 (15.5%)
Blood absoluteEx-smoker	47 (62.7%)	40 (54.1%)	9 (60.0%)	56 (58.9%)	37 (43.0%)	93 (51.4%)
Blood absoluteCurrent smoker	21 (28.0%)	25 (33.8%)	5 (33.3%)	28 (29.5%)	32 (37.2%)	60 (33.1%)
Post- bronchodilator test FEV1/FVC (%)	52.8 ± 8.9	52.9 ± 8.0	52.7 ± 5.8	53.1 ± 8.4	53.9 ± 8.6	53.5 ± 8.5
Airflow limitation severity (GOLD spirometric classification)						
I	3 (4.0%)	2 (2.7%)	1 (6.7%)	3 (3.2%)	15 (17.4%)	18 (9.9%)
II	40 (53.3%)	34 (45.9%)	8 (53.3%)	53 (55.8%)	56 (65.1%)	109 (60.2%)
III	28 (37.3%)	33 (44.6%)	5 (33.3%)	34 (35.8%)	15 (17.4%)	49 (27.1%)
IV	4 (5.3%)	5 (6.8%)	1 (6.7%)	5 (5.3%)	0 (0.0%)	5 (2.8%)
Laboratory findings						
WBC (10^9^/L)	11.4 ± 4.3	12.0 ± 4.7	12.8 ± 5.2	11.6 ± 4.6	9.5 ± 3.3	10.6 ± 4.2
Blood eosinophil percentage > 2%	34 (45.3%)	25 (33.8%)	5 (33.3%)	39 (41.1%)	38 (44.2%)	77 (42.5%)
Blood eosinophil percentage > 4%	11 (14.7%)	8 (10.8%)	1 (6.7%)	13 (13.7%)	18 (20.9%)	31 (17.1%)
Blood absolute eosinophil count > 150 cells/μL	37 (49.3%)	33 (44.6%)	9 (60.0%)	45 (47.4%)	46 (53.5%)	91 (50.3%)
Blood absolute eosinophil count >300 cells/μL	20 (26.7%)	15 (20.3%)	1 (6.7%)	23 (24.2%)	21 (24.4%)	44 (24.3%)

Abbreviations: FEV1, forced expiratory volume in one second; FVC, forced vital capacity; GOLD, Global Initiative for Chronic Obstructive Lung Disease; RCC, respiratory care center; WBC, white blood count.

### Associations between airflow limitation severity and short-term in-RCC adverse outcomes and between blood eosinophil level and short-term in-RCC adverse outcomes

**Figure 2:**
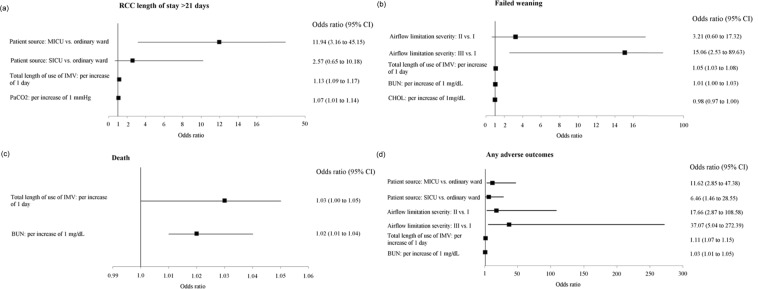
The factors associated with short-term in-respiratory care center adverse treatment outcomes. Abbreviations: BUN, blood urea nitrogen; CHOL, cholesterol; CI, confidence interval; IMV, invasive mechanical ventilation; MICU, medical intensive care unit; PaCO2, partial pressure of carbon dioxide; RCC, respiratory care center; SICU, surgical intensive care unit; vs., versus.

Overall, 41.4% (75/181), 40.9% (74/181), 8.3% (15/181), and 52.5% (95/181) of the participants had a prolonged RCC admission (RCC length of stay >21 days), failed weaning, died, and any adverse outcomes of interest, respectively (see Table 1). Cut-off values for blood eosinophilia were analyzed in this study (>2% versus ≤2%, >4% versus ≤4%, >150 cells/μL versus ≤150 cells/μL, and >300 cells/μL versus ≤300 cells/μL). Of note, simple and multiple logistic regression analyses show a moderate (the Global Initiative for Chronic Obstructive Lung Disease [GOLD] II) and /or severe (GOLD III) airflow limitation were significantly associated with short-term in-RCC adverse outcomes in terms of failed weaning (for GOLD III versus GOLD I, OR: 15.06, 95% CI: 2.53–89.63) and having any adverse outcomes of interest (for GOLD II versus GOLD I, OR: 17.66, 95% CI: 2.87–108.58; for GOLD III versus GOLD I, OR: 37.07, 95% CI: 5.04–272.39), while blood eosinophilia defined by any cut-off value in this study was not associated with any short-term in-RCC adverse outcomes (see Supplementary Tables S2, S3 and Fig. 2a–d).

### Sensitivity analyses for the impact on the factors associated with short-term in-RCC adverse outcomes by change in assumption

We performed sensitivity analyses on each short-term in-RCC adverse treatment outcome of interest with post-bronchodilator test FEV1% predicted, a continuous variable for airflow limitation severity, and various definitions of blood eosinophilia for patients with and without a diagnosis of asthma and unreasonable outliers and resulted in the same conclusions (see Supplementary Tables S2–S5).

## Discussion

It is the first that our study clarifies clinical insights into the role of severity of lung function and blood eosinophil value in patients with COPD and PMV. The most important finding is that, instead of blood eosinophilia, GOLD II and/or III airflow limitation was an independent risk factor for short-term in-RCC adverse outcomes in terms of failed weaning and having any adverse outcomes of interest.

The GOLD committee in 2011 moved away from a linear, one-dimensional classification of severity groups, defined only by degree of airflow limitation (FEV1% predicted), to a two-dimensional assessment that takes into consideration both exacerbation risk and symptom assessment. The exacerbation risk of COPD is determined by exacerbation history in the previous year and spirometric classification of airflow limitation by the GOLD grade^15^. A body of evidence has indicated that the addition of assessment of symptoms and history of exacerbation within one year to severity of airflow limitation does not provide better prognostic value on COPD outcomes regarding hospitalizations, mortality, and time to death though it identifies more patients with COPD at high risk of exacerbation^16–18^. Together with our finding that airflow limitation severity as determined by GOLD spirometric classification was an independent risk factor for short-term in-RCC adverse treatment outcomes in terms of failed weaning and having any adverse outcomes of interest in patients with COPD and PMV, FEV1% predicted solely is still an useful and reliable parameter associated with clinical outcomes and should be taken into consideration when managing patients with COPD.

Peripheral blood eosinophil level may be an useful indicator of treatment outcomes when managing patients with COPD, as a number of studies have demonstrated associations between blood eosinophilia and a lower risk of pneumonia, a shorter length of hospital stay, and better quality of life and survival in patients with stable COPD. Furthermore, these studies have also demonstrated associations between blood eosinophilia and a shorter ICU length of stay in patients with COPD and community-acquired pneumonia that require admission to an ICU and support by invasive mechanical ventilation^19–22^. However, we found that blood eosinophilia using 2%, 4%, 150 cells/μL, and 300 cells/μL as thresholds was not associated with any short-term in-RCC treatment outcomes in this study. Taken together, this indicates that an unclear but complex interaction exists between eosinophils and COPD, and further studies are needed to investigate this issue.

We found that airflow limitation severity was robustly predictive of failed weaning and having any adverse outcomes of interest. This may arise from that the present study recorded the spirometric data within two years prior to the study admission, making it better reflect the participants’ current lung functions and forecast the outcomes. Moreover, compared to those from ordinary wards, participants transferred from ICUs had more severe illnesses and nutritional and metabolic problems and more use of sedatives. This costs patients from ICUs more time on the multi-disciplinary rehabilitation treatment and makes patient source be strongly associated with prolonged RCC admission and having any adverse outcomes of interest.

Previous studies have reported successful weaning rates of 75.0% (39/52) to 95.5% (64/67) in patients with COPD and PMV^11,23^, compared to 59.1% (107/181) in the current study. The lower weaning rate in our study may be due to different study designs and weaning protocols between studies. For example, the rates of non-invasive positive pressure ventilation after liberation from invasive mechanical ventilation (29.8% vs. 59.7%) and tracheostomized ventilation (64.6% vs. 100%) were lower in the current study^11^, although one report showed that the method of invasive mechanical ventilation (tracheostomy vs. endo-tracheal tube) was not associated with weaning outcomes in patients with PMV in a RCC^24^.

All of the studied participants received a standardized and consistent in-RCC hospital treatment course, including the same physician being in charge throughout the study period, the multi-disciplinary rehabilitation treatment and respiratory therapist-implemented ventilator weaning protocol, and one type of mechanical ventilator, reducing bias in treatment outcomes. In addition, all of the participants were confirmed to have the diagnosis of COPD by spirometric data, ensuring a valid study population of patients with COPD. All the strengths of this study compensate for an important limitation of this study that a low ratio of RCC beds to ICU beds may have led to bias with regards to patient selection.

In this study, we found that the severity of airflow limitation defined based on GOLD grades was an independent factor associated with short-term in-RCC treatment outcomes in terms of mechanical ventilator weaning and presence of any adverse outcomes of interest^6^. Previously, we also found that blood eosinophil level was significantly associated with the in-ICU treatment outcome of ICU length of stay^22^. Taken together, this information provides evidence that these two biomarkers have predictive value and are helpful for physicians when managing patients with COPD and PMV and those critically ill with COPD. Further studies should enroll a larger number of patients to validate our findings and explore the factors associated with long-term in-RCC treatment outcomes.

## Conclusions

The severity of airflow limitation as measured by FEV1% predicted rather than the blood eosinophil level was an independent factor for short-term in-RCC treatment outcomes in terms of mechanical ventilator weaning and presence of any adverse outcomes of interest in this study. These findings have important clinical implications and should be considered in the management of patients with COPD and PMV.

## Methods

### Study design, setting and population

This retrospective cross-sectional study included patients with COPD and PMV admitted to the RCC of Taichung Veterans General Hospital located in central Taiwan between January 2010 and December 2015. The study facility was a 12-bed RCC offering a specialist weaning service for patients with PMV within a 1412-bed tertiary referral hospital containing 136 ICU beds. The pulmonary and critical care specialist served as the primary physician for all patients throughout the study period. The diagnosis of COPD was confirmed spirometrically according to GOLD 2017 strategy for all patients^6^. PMV was defined as continuous mechanical ventilatory support for respiratory failure due to any reason for at least 14 days. Patients aged <40 years old, those with a history of asthma, and, for the study purpose, those without any spirometric recordings in the past two years were excluded from this study. Furthermore, only the first admission was included for the patients with multiple RCC admissions that fulfilled all of the inclusion and exclusion criteria during the study period to address possible bias toward allocation of patients to certain types of treatment outcomes of interest arising from multiple admissions to the RCC in the same patient. The Institutional Review Board and Ethics Committee of Taichung Veterans General Hospital approved this study (approval number: CE14351A), waived the need for informed consent from the participants because the study was based on a retrospective electronic medical chart review, and confirmed that all methods were performed in accordance with the relevant guidelines and regulations.

### Data collection and definitions of study groups

A detailed patient record form was completed for each participant by reviewing and recording clinical data from electronic medical records throughout the study hospitalization. To explore the factors associated with short-term in-RCC adverse treatment outcomes of interest, the participants were categorized into those with an RCC length of stay >21 days, those who failed weaning and died in the RCC, those with any adverse treatment outcomes of interest to address competing risk of adverse outcomes against each other, and those without any adverse treatment outcomes of interest as controls for comparison. Further details are provided in the Supplementary Methods S1.

### RCC weaning process and definitions of weaning outcomes

During the study period, consistent multi-disciplinary rehabilitation treatment and respiratory therapist-implemented ventilator weaning protocol were applied, and mechanical ventilation was implemented based on the standards of the RCC at the study institute. Successful weaning was defined as liberation from invasive mechanical ventilatory support on discharge from the RCC. Otherwise, the case was defined as failure to wean (see Supplementary Methods S2 for further details).

### Statistical analysis

All data were expressed as number (percentage) for categorical variables or mean and standard deviation for continuous variables. Extreme values were considered to be outside the boundaries with 75% of the sample dataset +3.0× interquartile range or 25% of the sample dataset −3.0× interquartile range and were excluded from analysis only if it was unreasonable or even impossible for certain types of data^25,26^. All of the available data were analyzed in cases where some data were missing. For univariate analysis, comparisons were conducted using the independent t-test for continuous variables and the chi-square test for categorical variables. Simple and multiple logistic regression models were used to analyze associated factors for various short-term in-RCC clinical outcomes if they were significant in univariate analysis with and without missing data. In comparisons of independent variables, odds ratios and 95% confidence intervals were obtained. Statistical significance was set at p < 0.05. Statistical analysis was performed using SPSS software version 18.0 (SPSS Inc., Chicago, IL, USA).

## Supplementary information

The association between airflow limitation and blood eosinophil levels with treatment outcomes in patients with chronic obstructive pulmonary disease and prolonged mechanical ventilation

## Data Availability

No additional unpublished data from the study are available.
