# Supplementary material for: The association between airflow limitation and blood eosinophil levels with treatment outcomes in patients with chronic obstructive pulmonary disease and prolonged mechanical ventilation
Source: Sci Rep. 2019 Sep 17;9:13420. doi: 10.1038/s41598-019-49918-z (PMC6748958; doi:10.1038/s41598-019-49918-z)
Supplement: Supplementary file 1 — The association between airflow limitation and blood eosinophil levels with treatment outcomes in patients with chronic obstructive pulmonary disease and prolonged mechanical ventilation [file 41598_2019_49918_MOESM1_ESM.pdf]

SUPPLEMENTARY INFORMATION

TITLE: The association between airflow limitation and blood eosinophil levels with treatment outcomes in patients with chronic obstructive pulmonary disease and prolonged mechanical ventilation

Authors' full names: Wei-Chang Huang MD, Chen-Cheng Huang MD, Pi-Chu Wu MD, Chao-Jung Chen MD, Ya-Hua Cheng BS, Hui-Chen Chen MD, Ching-Hsiao Lee PhD, Ming-Feng Wu PhD, Jeng-Yuan Hsu PhD

Supplementary Methods S1- Data collection and definitions of study groups

For each participant, a detailed patient record form was completed which included age, gender, body mass index, smoking history, patient source, reasons for invasive mechanical ventilation (IMV), the spirometry results within two years prior to this admission as performed and interpreted based on the American Thoracic Society statement, which defines a positive bronchodilator test as forced expiratory volume in one second or forced vital capacity improvement from a pre-dose value by ≥ 12% and ≥ 200 ml,[1] co-morbidities, and lengths of hospital stay, hospital stay before transfer to the respiratory care center (RCC) and use of IMV before transfer to the RCC. In addition, modified Glasgow Coma Scale with verbal score as one,[2] Acute Physiology and Chronic Health Evaluation II score, initial laboratory findings upon arrival at the RCC, the type of chronic obstructive pulmonary disease medications, rapid shallow index, the type of artificial airway, ventilator settings upon arrival at the RCC, and whether or not the participants used non-invasive positive pressure ventilation after successful liberation from IMV support during the RCC stay were also recorded. The in-RCC hospital course with the short-term treatment outcomes of interest including RCC length of stay, weaning outcomes, and death were also recorded. All patient information was anonymized and de-identified prior to analysis. All available data were analyzed after allowing for missing information, although extreme data were removed from this study.

References

1 Lung function testing: selection of reference values and interpretative strategies. American Thoracic Society. *Am. Rev. Res.p Dis.* 1991; **144**: 1202-18.

2 Huang WC, Wu PC, Chen CJ, Cheng YH, Shih SJ, Chen HC, Wu CL. High-frequency chest wall oscillation in prolonged mechanical ventilation patients: a randomized controlled

trial. *Clin. Respir. J.* 2016; **10**: 272-81.

Supplementary Methods S2- RCC weaning process and definitions of weaning outcomes

All of the participants received multi-disciplinary rehabilitation treatment that included conventional chest percussion and nutritional and physiotherapy consultations for individualized nutrition formulas and exercise programs. The study unit used only one type of mechanical ventilator - the Covidien Puritan Bennett™ 840 ventilator. A respiratory therapist-implemented weaning protocol was started when the participant met all of the following criteria: causes of respiratory failure solved; no evidence of hemodynamic instability; no use of inotropic agents; systolic blood pressure >100 mmHg; heart rate < 110 and > 50 bpm; body temperature < 38°C; fraction of inspired oxygen < 40%; and PEEP ≤ 8 mmHg. The protocol was composed of six steps (steps 0-5), and the patients progressed one step every 24 hours from full ventilatory support (step 0) to spontaneous, unassisted breathing (step 5). Ventilator support was decreased gradually by reducing the level of pressure support ventilation with a 2-cmH2O decrement in each step, from 14 to 10 cmH2O, followed by a spontaneous T-piece breathing trial. All of these processes were only conducted during the daytime (from 8 a.m. to 6 p.m.) with full ventilator support being applied at night (from 6 p.m. to 8 a.m.). Patients were liberated from mechanical ventilatory support after passing the spontaneous T-piece breathing trial during which no indicator of weaning failure was present.

Supplementary Table S1. Demographic and clinical characteristics of all participants and study groups categorized by respiratory care center length of stay, weaning outcomes, and mortality.

	Individuals with RCC length of stay > 21 days (n=75)	p value	Individuals with failed weaning (n=74)	p value	Individuals with death (n=15)	p value	Individuals with any adverse outcomes (n= 95)	p value	Individuals without any adverse outcomes (n= 86)	Total (n=181)
Age (years)	78.0±10.4	0.017*	77.7±11.3	0.032*	76.4±14.0	0.442	77.1±11.2	0.048*	73.5±13.1	75.4±12.3
Male gender	65 (86.7%)	0.617	67 (90.5%)	0.218	14 (93.3%)	0.454	84 (88.4%)	0.362	71 (82.6%)	155 (85.6%)
BMI		0.242		0.272		0.523		0.356		
Available number	73 (97.3%)		73 (98.6%)		15 (100 %)		93 (97.9%)		85 (98.8%)	178 (98.3%)
Mean±SD	22.3±4.0		22.4±3.6		22.3±3.9		22.5±4.0		23.1±5.0	22.8±4.5
Smoking history		0.031*		0.279		0.352		0.082		
Never	7 (9.3%)		9 (12.2%)		1 (6.7%)		11 (11.6%)		17 (19.8%)	28 (15.5%)
Ex-smoker	47 (62.7%)		40 (54.1%)		9 (60.0%)		56 (58.9%)		37 (43.0%)	93 (51.4%)
Current smoker	21 (28.0%)		25 (33.8%)		5 (33.3%)		28 (29.5%)		32 (37.2%)	60 (33.1%)
Patient source		0.002*		0.005*		0.099		0.009*		
MICU	51 (68.0%)		49 (66.2%)		9 (60.0%)		60 (63.2%)		35 (40.7%)	95 (52.5%)
SICU	15 (20.0%)		16 (21.6%)		6 (40.0%)		23 (24.2%)		31 (36.0%)	54 (29.8%)
Ordinary ward	9 (12.0%)		9 (12.2%)		0 (0.0%)		12 (12.6%)		20 (23.3%)	32 (17.7%)
Reasons for IMV		0.020*		0.038*		0.210		0.015*		
Infectious	46 (61.3%)		46 (62.2%)		10 (66.7%)		58 (61.1%)		33 (38.4%)	91 (50.3%)
Pneumonia	38 (50.7%)		39 (52.7%)		9 (60.0%)		49 (51.6%)		27 (31.4%)	76 (42.0%)
Urosepsis	4 (5.3%)		4 (5.4%)		0 (0.0%)		4 (4.2%)		2 (2.3%)	6 (3.3%)
Intra-abdominal infection	4 (5.3%)		3 (4.1%)		1 (6.7%)		5 (5.3%)		4 (4.7%)	9 (5.0%)
Non-infectious	29 (38.7%)		28 (37.8%)		5 (33.3%)		37 (38.9%)		53 (61.6%)	90 (49.7%)
Intracranial hemorrhage	5 (6.7%)		7 (9.5%)		2 (13.3%)		9 (9.5%)		17 (19.8%)	26 (14.4%)
Ischemic stroke	0 (0.0%)		0 (0.0%)		0 (0.0%)		0 (0.0%)		4 (4.7%)	4 (2.2%)
Cardiac disease	8 (10.7%)		7 (9.5%)		1 (6.7%)		9 (9.5%)		6 (7.0%)	15 (8.3%)
Chronic lung disease	5 (6.7%)		6 (8.1%)		0 (0.0%)		7 (7.4%)		7 (8.1%)	14 (7.7%)
Post-operation	0 (0.0%)		0 (0.0%)		0 (0.0%)		0 (0.0%)		3 (3.5%)	3 (1.7%)
Neurologic disease	2 (2.7%)		2 (2.7%)		0 (0.0%)		2 (2.1%)		5 (5.8%)	7 (3.9%)
Malignancy	3 (4.0%)		2 (2.7%)		1 (6.7%)		4 (4.2%)		3 (3.5%)	7 (3.9%)
Chronic kidney	3 (4.0%)		2 (2.7%)		1 (6.7%)		3 (3.2%)		0 (0.0%)	3 (1.7%)

disease										
Miscellaneous	3 (4.0%)		2 (2.7%)		0 (0.0%)		3 (3.2%)		8 (9.3%)	11 (6.1%)
Post- bronchodilator test FEV1/FVC (%)	52.8±8.9	0.423	52.9±8.0	0.438	52.7±5.8	0.504	53.1±8.4	0.514	53.9±8.6	53.5±8.5
Positive bronchodilator test[&]	14 (18.7%)	0.419	15 (20.3%)	0.287	0 (0.0%)	0.362	18 (18.9%)	0.355	11 (12.8%)	29 (16.0%)
Airflow limitation severity (GOLD spirometric classification)		0.000*		0.000*		0.035*		0.000*		
I	3 (4.0%)		2 (2.7%)		1 (6.7%)		3 (3.2%)		15 (17.4%)	18 (9.9%)
II	40 (53.3%)		34 (45.9%)		8 (53.3%)		53 (55.8%)		56 (65.1%)	109 (60.2%)
III	28 (37.3%)		33 (44.6%)		5 (33.3%)		34 (35.8%)		15 (17.4%)	49 (27.1%)
IV	4 (5.3%)		5 (6.8%)		1 (6.7%)		5 (5.3%)		0 (0.0%)	5 (2.8%)
Co-morbidity										
Ischemic heart disease	25 (33.3%)	0.111	22 (29.7%)	0.272	4 (26.7%)	0.735	29 (30.5%)	0.193	18 (20.9%)	47 (26.0%)
Congestive heart failure	25 (33.3%)	0.156	23 (31.1%)	0.268	4 (26.7%)	0.741	29 (30.5%)	0.265	19 (22.1%)	48 (26.5%)
Arrythmia	20 (26.7%)	0.751	20 (27.0%)	0.714	4 (26.7%)	0.750	24 (25.3%)	0.888	20 (23.3%)	44 (24.3%)
Chronic renal failure	28 (37.3%)	0.021*	25 (33.8%)	0.067	7 (46.7%)	0.043*	33 (34.7%)	0.037*	17 (19.8%)	50 (27.6%)
Malignancy	7 (9.3%)	0.797	5 (6.8%)	1.000	1 (6.7%)	1.000	8 (8.4%)	0.933	6 (7.0%)	14 (7.7%)
Length of hospital stay (days)	70.7±24.2	0.021*	64.4±25.5	0.353	64.0±31.1	0.664	67.2±25.6	0.103	60.2±31.7	63.8±28.8
Length of hospital stay before transfer to RCC (days)	24.8±10.2	0.946	25.1±11.4	0.918	29.3±13.2	0.201	25.1±11.2	0.938	24.9±11.8	25.0±11.5
Total length of use of IMV (days)	61.4±18.6	0.000*	57.5±20.7	0.000*	62.4±30.5	0.004*	57.2±20.0	0.000*	34.8±13.6	46.6±20.6
Length of use of IMV before transfer to RCC (days)	28.2±12.6	0.937	28.3±13.2	0.945	29.4±16.2	0.781	28.3±13.3	0.962	28.4±12.2	28.4±12.7
Modified GCS[#]	9.6±1.8	0.019*	9.7±1.8	0.053	9.4±1.3	0.043*	9.7±1.7	0.038*	10.2±1.4	10.0±1.6
APACHE II score	19.8±4.6	0.002*	19.6±4.4	0.004*	20.1±4.0	0.029*	19.6±4.6	0.003*	17.6±4.0	18.7±4.4
Laboratory findings										
WBC (10^9/L)	11.4±4.3	0.002*	12.0±4.7	0.000*	12.8±5.2	0.029*	11.6±4.6	0.000*	9.5±3.3	10.6±4.2
Blood eosinophil	34 (45.3%)	1.000	25 (33.8%)	0.238	5 (33.3%)	0.616	39 (41.1%)	0.783	38 (44.2%)	77 (42.5%)

percentage > 2%										
Blood eosinophil percentage > 4%	11 (14.7%)	0.409	8 (10.8%)	0.130	1 (6.7%)	0.291	13 (13.7%)	0.274	18 (20.9%)	31 (17.1%)
Blood absolute eosinophil count > 150 cells/μL	37 (49.3%)	0.713	33 (44.6%)	0.335	9 (60.0%)	0.852	45 (47.4%)	0.501	46 (53.5%)	91 (50.3%)
Blood absolute eosinophil count > 300 cells/μL	20 (26.7%)	0.884	15 (20.3%)	0.662	1 (6.7%)	0.180	23 (24.2%)	1.000	21 (24.4%)	44 (24.3%)
Hemoglobin (g/dL)	9.8±1.4	0.063	9.9±1.4	0.098	9.4±0.9	0.025*	9.9±1.4	0.086	10.2±1.3	10.0±1.4
Albumin (g/dL)	2.7±0.5	0.061	2.7±0.5	0.023*	2.7±0.6	0.200	2.8±0.5	0.089	2.9±0.4	2.8±0.5
BUN (mg/dL)		0.003*		0.000*		0.009*		0.000*		
Available number	69 (92.0%)		67 (90.5%)		13 (86.7%)		87 (91.6%)		73 (84.9%)	160 (88.4%)
Mean±SD	49.3±41.9		53.1±43.1		79.5±54.9		52.3±42.5		32.2±20.0	43.1±35.5
Creatinine (mg/dL)	1.8±1.7	0.008*	1.7±1.6	0.025*	1.8±1.6	0.143	1.7±1.6	0.007*	1.2±1.0	1.5±1.4
pH	7.5±0.1	0.068	7.5±0.1	0.097	7.5±0.0	0.412	7.5±0.1	0.110	7.5±0.1	7.5±0.1
PaCO2 (mmHg)	39.9±8.9	0.024*	39.4±9.2	0.064	40.6±11.7	0.254	39.4±8.9	0.046*	36.9±7.8	38.2±8.5
PaO2/FiO2 ratio	289.9±156.5	0.614	283.2±151.9	0.440	290.0±142.4	0.780	288.8±152.3	0.554	302.9±167.5	295.5±159.4
CHOL (mg/dL)		0.005*		0.001*		0.009*		0.001*		
Available number	73 (97.3%)		73 (98.6%)		15 (100 %)		93 (97.9%)		85 (98.8%)	178 (98.3%)
Mean±SD	122.2±35.8		118.1±37.2		110.6±32.0		120.3±36.7		139.2±39.0	129.3±38.9
Prealbumin (g/L)		0.013*		0.003*		0.003*		0.006*		
Available number	74 (98.7%)		73 (98.6%)		15 (100 %)		94 (98.9%)		86 (100%)	180 (99.4%)
Mean±SD	0.1±0.1		0.1±0.1		0.1±0.1		0.1±0.1		0.2±0.1	0.2±0.1
COPD medications										
Use of systemic steroids	0 (0.0%)	NA	0 (0.0%)	NA	0 (0.0%)	NA	0 (0.0%)	NA	0 (0.0%)	0 (0.0%)
Use of short-acting bronchodilators	75 (100%)	NA	74 (100%)	NA	15 (100%)	NA	95 (100%)	NA	86 (100%)	181 (100%)
RSI※		0.750		0.871		0.488		0.853		
Available number	44 (58.7%)		38 (51.4%)		5 (33.3%)		52 (54.7%)		51 (59.3%)	103 (56.9%)
Mean±SD	166.7±130.0		162.7±134.0		200.4±134.3		162.8±126.3		158.1±128.8	160.5±127.0
Artificial airway		0.709		0.894		0.998		0.977		
Tracheostomy	51 (68.0%)		49 (66.2%)		9 (60.0%)		62 (65.3%)		55 (64.0%)	117 (64.6%)
Endotracheal tube	24 (32.0%)		25 (33.8%)		6 (40.0%)		33 (34.7%)		31 (36.0%)	64 (35.4%)
Ventilator settings										
Mode		1.000		0.763		0.343		0.683		

Volume control	54 (72.0%)		55 (74.3%)		13 (86.7%)		71 (74.7%)		61 (70.9%)	132 (72.9%)
Pressure control	21 (28.0%)		19 (25.7%)		2 (13.3%)		24 (25.3%)		25 (29.1%)	49 (27.1%)
Tidal volume (ml/kg)	9.7±0.7	0.576	9.7±0.7	0.293	9.9±0.5	0.653	9.7±0.7	0.511	9.8±0.6	9.8±0.7
Respiratory rate (breaths per minute)	17.3±0.9	0.120	17.2±1.0	0.435	17.3±1.0	0.391	17.3±1.0	0.148	17.1±1.0	17.2±1.0
Minute ventilation (liters per minute)		0.339		0.153		0.414		0.387		
Available number	35 (46.7%)		29 (39.2%)		4 (26.7%)		42 (44.2%)		42 (48.8%)	84 (46.4%)
Mean±SD	7.9±3.6		8.4±3.5		8.7±5.1		7.8±3.5		7.1±3.5	7.5±3.5
Use of NIPPV after successful liberation from IMV support during the RCC stay	27 (36.0%)	0.051	31 (41.9%)	0.007*	5 (33.3%)	0.322	36 (37.9%)	0.020*	18 (20.9%)	54 (29.8%)

*p<0.05 when compared to Individuals without any adverse outcomes.

&Positive bronchodilator test was defined as FEV 1 or FVC improvement from pre-dose value by≧12% and≧200 mL.

#Modified GCS, verbal score as one.

※RSI was defined as the ratio of respiratory frequency to tidal volume (breaths/minute/liter).

Abbreviations: APACHE II score, Acute Physiology and Chronic Health Evaluation II score; BMI, body mass index; BUN, blood urea nitrogen; CHOL, cholesterol; COPD, chronic obstructive pulmonary disease; FEV1, forced expiratory volume in one second; FiO2, fractional inspired oxygen; FVC, forced vital capacity; GCS, Glasgow Coma Scale; GOLD, Global Initiative for Chronic Obstructive Lung Disease; IMV, invasive mechanical ventilation; MICU, medical intensive care unit; NA, not applicable; NIPPV, noninvasive positive pressure ventilation; PaCO2, partial pressure of carbon dioxide; PaO2, arterial oxygen partial pressure; RCC, respiratory care center; RSI, rapid shallow index; SD, standard deviation; SICU, surgical intensive care unit; WBC, white blood count.

Supplementary Table S2. Simple logistic regression analysis for the significant factors associated with short-term in-RCC adverse treatment outcomes.

Significant factors for adverse treatment outcomes	All participants (n=181)		Participants including those with asthma and unreasonable outliers (n=187)	
	Odds ratio (95% CI)	p value	Odds ratio (95% CI)	p value
**RCC length of stay >21 days**				
Age: per 1-year increase	1.03 (1.01 to 1.06)	0.019*	1.03 (1.00 to 1.06)	0.028*
Smoking: ex-smoker vs. never smoker	3.07 (1.19 to 7.90)	0.020*	3.19 (1.24 to 8.20)	0.016*
Smoking: current smoker vs. never smoker	1.62 (0.59 to 4.42)	0.350	1.54 (0.56 to 4.19)	0.402
Patient source: MICU vs. ordinary ward	2.96 (1.24 to 7.07)	0.014*	3.14 (1.33 to 7.44)	0.009*
Patient source: SICU vs. ordinary ward	0.98 (0.37 to 2.60)	0.972	1.07 (0.41 to 2.79)	0.895
Reasons for invasive mechanical ventilation: Infectious vs. non-infectious	2.15 (1.18 to 3.93)	0.013*	2.19 (1.21 to 3.97)	0.010*
Airflow limitation severity: II vs. I	2.90 (0.79 to 10.63)	0.108	2.85 (0.78 to 10.42)	0.114
Airflow limitation severity: III vs. I	6.67 (1.71 to 26.04)	0.006*	7.14 (1.84 to 27.81)	0.005*
Airflow limitation severity: IV vs. I	20.00 (1.61 to 247.98)	0.020*	20.00 (1.61 to 247.98)	0.020*

Chronic renal failure: yes vs. no	2.28 (1.17 to 4.41)	0.015*	2.34 (1.22 to 4.51)	0.011*
Length of hospital stay: per increase of 1 day	1.02 (1.00 to 1.03)	0.011*	1.02 (1.00 to 1.03)	0.010*
Total length of use of IMV: per increase of 1 day	1.10 (1.07 to 1.14)	0.000*	1.11 (1.07 to 1.14)	0.000*
Modified GCS[#]: per increase of 1 point	0.79 (0.65 to 0.96)	0.017*	0.81 (0.67 to 0.98)	0.029*
APACHE II score: per increase of 1 point	1.10 (1.03 to 1.18)	0.006*	1.10 (1.02 to 1.17)	0.010*
WBC: per increase of 1000/μL	1.00 (1.00 to 1.00)	0.031*	1.00 (1.00 to 1.00)	0.037*
BUN: per increase of 1 mg/dL	1.01 (1.00 to 1.02)	0.063	1.01 (1.00 to 1.02)	0.069
Creatinine: per increase of 1 mg/dL	1.33 (1.06 to 1.67)	0.014*	1.32 (1.06 to 1.66)	0.015*
PaCO2: per increase of 1 mmHg	1.04 (1.00 to 1.08)	0.028*	1.04 (1.00 to 1.08)	0.033*
CHOL: per increase of 1mg/dL	0.99 (0.98 to 1.00)	0.044*	0.99 (0.98 to 1.00)	0.049*
Prealbumin: per decrease of 0.1 g/L	0.02 (0.00 to 1.49)	0.077	0.06 (0.00 to 3.00)	0.156
**Failed weaning**				
Age: per 1-year increase	1.03 (1.00 to 1.06)	0.037*	1.03 (1.00 to 1.05)	0.049*
Patient source: MICU vs. ordinary ward	2.72 (1.14 to 6.49)	0.024*	2.89 (1.22 to 6.86)	0.016*
Patient source: SICU vs. ordinary ward	1.08 (0.41 to 2.83)	0.882	1.16 (0.45 to 3.02)	0.757

Reasons for invasive mechanical ventilation: infectious vs. non-infectious	2.26 (1.23 to 4.15)	0.008*	2.30 (1.27 to 4.18)	0.006*
Airflow limitation severity: II vs. I	3.63 (0.79 to 16.66)	0.098	3.59 (0.78 to 16.46)	0.100
Airflow limitation severity: III vs. I	16.50 (3.38 to 80.64)	0.001*	17.50 (3.59 to 85.35)	0.000*
Airflow limitation severity: IV vs. I	- (0.00 to -)	0.999	- (0.00 to -)	0.999
Total length of use of IMV: per increase of 1 day	1.06 (1.04 to 1.08)	0.000*	1.06 (1.04 to 1.08)	0.000*
APACHE II score: per increase of 1 point	1.08 (1.01 to 1.16)	0.023*	1.08 (1.01 to 1.15)	0.036*
WBC: per increase of 1000/μL	1.00 (1.00 to 1.00)	0.000*	1.00 (1.00 to 1.00)	0.000*
Albumin: per increase of 1 g/dL	0.42 (0.22 to 1.08)	0.060	0.89 (0.58 to 1.37)	0.592
BUN: per increase of 1 mg/dL	1.02 (1.00 to 1.03)	0.005*	1.01 (1.00 to 1.03)	0.006*
Creatinine: per increase of 1 mg/dL	1.19 (0.96 to 1.48)	0.109	1.19 (0.96 to 1.47)	0.116
CHOL: per increase of 1mg/dL	0.99 (0.98 to 1.00)	0.002*	0.99 (0.98 to 1.00)	0.003*
Prealbumin: per increase of 1 g/L	0.00 (0.00 to 0.13)	0.004*	0.01 (0.00 to 0.31)	0.012*
Use of NIPPV after successful liberation from IMV support during the RCC stay: yes vs. no	2.63 (1.37 to 5.06)	0.004*	2.42 (1.28 to 4.57)	0.006*

Death

Airflow limitation severity: II vs. I	1.35 (0.16 to 11.46)	0.785	1.30 (0.15 to 11.02)	0.813
Airflow limitation severity: III vs. I	1.93 (0.21 to 17.77)	0.561	1.85 (0.20 to 16.98)	0.587
Airflow limitation severity: IV vs. I	4.25 (0.22 to 83.52)	0.341	4.25 (0.22 to 83.52)	0.341
Chronic renal failure: yes vs. no	2.50 (0.86 to 7.31)	0.094	2.55 (0.87 to 7.43)	0.087
Total length of use of IMV: per increase of 1 day	0.97 (0.95 to 0.99)	0.004*	1.03 (1.01 to 1.06)	0.004*
Modified GCS[#]: per increase of 1 point	0.83 (0.63 to 1.09)	0.172	0.82 (0.63 to 1.08)	0.160
APACHE II score: per increase of 1 point	1.08 (0.97 to 1.21)	0.180	1.08 (0.97 to 1.21)	0.164
WBC: per increase of 1000/μL	1.00 (1.00 to 1.00)	0.036*	1.00 (1.00 to 1.00)	0.033*
Hemoglobin: per increase of 1 g/dL	0.66 (0.42 to 1.04)	0.071	0.65 (0.41 to 1.03)	0.068
BUN: per increase of 1 mg/dL	1.02 (1.01 to 1.03)	0.001*	1.02 (1.01 to 1.03)	0.001*
CHOL: per increase of 1mg/dL	0.98 (0.96 to 1.00)	0.050	0.98 (0.97 to 1.00)	0.051
Prealbumin: per increase of 1 g/L	0.00 (0.00 to 0.08)	0.012*	0.00 (0.00 to 0.00)	0.012*

Any adverse outcomes

Age: per 1-year increase	1.03 (1.00 to 1.05)	0.051	1.02 (1.00 to 1.05)	0.070
Patient source: MICU vs. ordinary ward	2.86 (1.25 to 6.54)	0.013*	3.01 (1.33 to 6.84)	0.008*
Patient source: SICU vs. ordinary ward	1.24 (0.51 to 3.03)	0.642	1.31 (0.54 to 3.18)	0.547
Reasons for invasive mechanical ventilation: infectious vs. non-infectious	2.52 (1.38 to 4.58)	0.003*	2.55 (1.42 to 4.61)	0.002*
Airflow limitation severity: II vs. I	4.73 (1.30 to 17.28)	0.019*	4.58 (1.26 to 16.68)	0.021*
Airflow limitation severity: III vs. I	11.33 (2.85 to 45.07)	0.001*	12.00 (3.02 to 47.61)	0.000*
Airflow limitation severity: IV vs. I	- (0.00 to -)	0.999	- (0.00 to -)	0.999
Chronic renal failure: yes vs. no	2.16 (1.10 to 4.26)	0.026*	2.25 (1.15 to 4.41)	0.018*
Total length of use of IMV: per increase of 1 day	1.09 (1.06 to 1.12)	0.000*	1.10 (1.07 to 1.13)	0.000*
Modified GCS[#]: per increase of 1 point	0.81 (0.66 to 1.01)	0.054	0.83 (0.68 to 1.01)	0.067
APACHE II score: per increase of 1 point	1.11 (1.03 to 1.19)	0.004*	1.10 (1.03 to 1.18)	0.007*
WBC: per increase of 1000/µL	1.00 (1.00 to 1.00)	0.001*	1.00 (1.00 to 1.00)	0.001*
BUN: per increase of 1 mg/dL	1.02 (1.01 to 1.04)	0.001*	1.02 (1.01 to 1.04)	0.001*
Creatinine: per increase of 1 mg/dL	1.38 (1.08 to 1.77)	0.011*	1.38 (1.08 to 1.77)	0.011*
PaCO2: per increase of 1 mmHg	1.04 (1.01 to 1.08)	0.053	1.04 (1.00 to 1.07)	0.053

CHOL: per increase of 1mg/dL	0.99 (0.98 to 1.00)	0.002*	0.99 (0.98 to 1.00)	0.003*
Prealbumin: per increase of 1 g/L	0.00 (0.00 to 0.22)	0.007*	0.01 (0.00 to 0.47)	0.020*
Use of NIPPV after successful liberation from IMV support during the RCC stay: yes vs. no	2.31 (1.19 to 4.48)	0.014*	2.09 (1.10 to 3.98)	0.025*

*Statistically significant ($p<0.05$).

#Modified GCS, verbal score as one.

Abbreviations: CI, confidence interval; vs., versus; also see Supplementary Table S1.

Supplementary Table S3. Multiple logistic regression analysis for the independent factors associated with short-term in-RCC adverse treatment outcomes.

Significant factors for adverse treatment outcomes	All participants (n=181)		Participants including those with asthma and unreasonable outliers (n=187)	
	Odds ratio (95% CI)	p value	Odds ratio (95% CI)	p value
**RCC length of stay >21 days**				
Patient source: MICU vs. ordinary ward	11.94 (3.16 to 45.15)	0.000*	15.47 (3.94 to 60.76)	0.000*
Patient source: SICU vs. ordinary ward	2.57 (0.65 to 10.18)	0.180	4.17 (0.93 to 16.94)	0.056
Total length of use of IMV: per increase of 1 day	1.13 (1.09 to 1.17)	0.000*	1.13 (1.09 to 1.18)	0.000*
PaCO2: per increase of 1 mmHg	1.07 (1.01 to 1.14)	0.015*	1.07 (1.02 to 1.14)	0.013*
**Failed weaning**				
Airflow limitation severity: II vs. I	3.21 (0.60 to 17.32)	0.174	4.21 (0.75 to 23.59)	0.102
Airflow limitation severity: III vs. I	15.06 (2.53 to 89.63)	0.003*	22.88 (3.65 to 143.59)	0.001*
Total length of use of IMV: per increase of 1 day	1.05 (1.03 to 1.08)	0.000*	1.05 (1.03 to 1.08)	0.000*
BUN: per increase of 1 mg/dL	1.01 (1.00 to 1.03)	0.046*	1.01 (1.00 to 1.03)	0.047*

CHOL: per increase of 1mg/dL	0.98 (0.97 to 1.00)	0.008*	0.99 (0.98 to 1.00)	0.030*
**Death**				
Total length of use of IMV: per increase of 1 day	1.03 (1.00 to 1.05)	0.058	1.03 (1.00 to 1.06)	0.056
BUN: per increase of 1 mg/dL	1.02 (1.01 to 1.04)	0.002*	1.02 (1.01 to 1.04)	0.001*
**Any adverse outcomes**				
Patient source: MICU vs. ordinary ward	11.62 (2.85 to 47.38)	0.001*	12.33 (3.04 to 50.03)	0.000*
Patient source: SICU vs. ordinary ward	6.46 (1.46 to 28.55)	0.014*	6.62 (1.52 to 28.72)	0.012*
Airflow limitation severity: II vs. I	17.66 (2.87 to 108.58)	0.002*	17.70 (2.84 to 110.34)	0.002*
Airflow limitation severity: III vs. I	37.07 (5.04 to 272.39)	0.000*	42.85 (5.76 to 318.63)	0.000*
Total length of use of IMV: per increase of 1 day	1.11 (1.07 to 1.15)	0.000*	1.11 (1.07 to 1.16)	0.000*
BUN: per increase of 1 mg/dL	1.03 (1.01 to 1.05)	0.008*	1.03 (1.01 to 1.05)	0.005*

*Statistically significant (p<0.05).
Abbreviations: see Supplementary Tables S1 and S2.

Supplementary Table S4. Associations between airflow limitation severity and blood eosinophil levels with short-term in-RCC adverse outcomes for all participants.

	Individuals with RCC length of stay > 21 days (n=75)	p value	Individuals with failed weaning (n=74)	p value	Individuals with death (n=15)	p value	Individuals with any adverse outcomes (n= 95)	p value	Individuals without any adverse outcomes (n= 86)	Total (n=181)
**Airflow limitation severity**										
Post- bronchodilator test FEV1 % predicted	53.4±15.1	0.000*	49.8±13.6	0.000*	50.7±14.1	0.003*	52.8±14.0	0.000*	63.0±14.6	57.7±15.1
**Blood eosinophil levels**										
Blood eosinophil percentage (%)	2.4±2.9	0.626	1.9±2.9	0.147	1.5±1.4	0.130	2.2±3.0	0.406	2.6±2.8	2.4±2.9
Blood absolute eosinophil count (cells/μL)	248.3±293.7	0.713	197.8±237.0	0.418	165.9±139.7	0.380	228.9±283.7	0.949	2321.5±281.2	230.1±281.8
Blood eosinophil percentage >3%	19 (25.3%)	0.607	13 (17.6%)	0.094	1 (6.7%)	0.064	21 (22.1%)	0.282	26 (30.2%)	47 (26.0%)
Blood absolute eosinophil count >200 cells/μL	34 (45.3%)	0.892	28 (37.8%)	0.614	7 (46.7%)	1.000	40 (42.1%)	1.000	37 (43.0%)	77 (42.5%)
Blood absolute eosinophil count >400 cells/μL	16 (21.3%)	0.217	11 (14.9%)	0.881	1 (6.7%)	0.688	17 (17.9%)	0.458	11 (12.8%)	28 (15.5%)
Blood eosinophil percentage > 2% or absolute count >200 cells/μL	38 (50.7%)	0.825	30 (40.5%)	0.456	7 (46.7%)	1.000	44 (46.3%)	0.973	41 (47.7%)	85 (47.0%)
Blood eosinophil percentage >3% or absolute count >300 cells/μL	23 (30.7%)	0.580	16 (21.6%)	0.068	2 (13.3%)	0.134	26 (27.4%)	0.274	31 (36.0%)	57 (31.5%)
Blood eosinophil percentage >4% or	18 (24.0%)	1.000	14 (18.9%)	0.635	2 (13.3%)	0.513	20 (21.1%)	0.859	20 (23.3%)	40 (22.1%)

absolute count >400 cells/μL	

*p<0.05 when compared to Individuals without any adverse outcomes.

Abbreviations: see Supplementary Table S1.

Supplementary Table S5. Demographic and clinical characteristics of participants including those with asthma and unreasonable outliers and study groups categorized by respiratory care center length of stay, weaning outcomes, and mortality.

	Individuals with RCC length of stay > 21 days (n=78)	p value	Individuals with failed weaning (n=77)	p value	Individuals with death (n=15)	p value	Individuals with any adverse outcomes (n= 98)	p value	Individuals without any adverse outcomes (n= 89)	Total (n=187)
Age (years)	77.9±10.5	0.029*	77.6±11.3	0.047*	76.4±14.0	0.478	77.0±11.3	0.049*	73.8±13.1	75.5±12.2
Male gender	68 (87.2%)	0.609	70 (90.9%)	0.214	14 (93.3%)	0.457	87 (88.8%)	0.368	74 (83.1%)	161 (86.1%)
BMI		0.247		0.276		0.570		0.374		
Available number	76 (97.4%)		76 (98.7%)		15 (100 %)		96 (98.0%)		88 (98.9%)	184 (98.4%)
Mean±SD	22.2±4.0		22.3±3.6		22.3±3.9		22.4±4.0		23.0±4.9	22.7±4.5
Smoking history		0.017*		0.193		0.354		0.051		
Never	7 (9.0%)		9 (11.7%)		1 (6.7%)		11 (11.2%)		17 (19.1%)	28 (15.0%)
Ex-smoker	50 (64.1%)		43 (55.8%)		9 (60.0%)		59 (60.2%)		38 (42.7%)	97 (51.9%)
Current smoker	21 (26.9%)		25 (32.5%)		5 (33.3%)		28 (28.6%)		34 (38.2%)	62 (33.2%)
Patient source		0.002*		0.004*		0.094		0.006*		
MICU	53 (67.9%)		51 (66.2%)		9 (60.0%)		62 (63.3%)		36 (40.4%)	98 (52.4%)
SICU	16 (20.5%)		17 (22.1%)		6 (40.0%)		24 (24.5%)		32 (36.0%)	56 (29.9%)
Ordinary ward	9 (11.5%)		9 (11.7%)		0 (0.0%)		12 (12.2%)		21 (23.6%)	33 (17.6%)
Reasons for IMV		0.018*		0.033*		0.200		0.015*		
Infectious	48 (61.5%)		48 (62.3%)		10 (66.7%)		60 (61.2%)		34 (38.2%)	94 (50.3%)
Pneumonia	40 (51.3%)		41 (53.2%)		9 (60.0%)		51 (52.0%)		27 (30.3%)	78 (41.7%)
Urosepsis	4 (5.1%)		4 (5.2%)		0 (0.0%)		4 (4.1%)		2 (2.2%)	6 (3.2%)
Intra-abdominal infection	4 (5.1%)		3 (3.9%)		1 (6.7%)		5 (5.1%)		5 (5.6%)	10 (5.3%)
Non-infectious	30 (38.5%)		29 (37.7%)		5 (33.3%)		38 (38.8%)		55 (61.8%)	93 (49.7%)
Intracranial hemorrhage	5 (6.4%)		7 (9.1%)		2 (13.3%)		9 (9.2%)		17 (19.1%)	26 (13.9%)
Ischemic stroke	0 (0.0%)		0 (0.0%)		0 (0.0%)		0 (0.0%)		4 (4.5%)	4 (2.1%)
Cardiac disease	8 (10.3%)		7 (9.1%)		1 (6.7%)		9 (9.2%)		6 (6.7%)	15 (8.0%)
Chronic lung disease	5 (6.4%)		6 (7.8%)		0 (0.0%)		7 (7.1%)		8 (9.0%)	15 (8.0%)
Post-operation	0 (0.0%)		0 (0.0%)		0 (0.0%)		0 (0.0%)		3 (3.4%)	3 (1.6%)

Neurologic disease	2 (2.6%)		2 (2.6%)		0 (0.0%)		2 (2.0%)		5 (5.6%)	7 (3.7%)
Malignancy	3 (3.8%)		2 (2.6%)		1 (6.7%)		4 (4.1%)		4 (4.5%)	8 (4.3%)
Chronic kidney disease	3 (3.8%)		2 (2.6%)		1 (6.7%)		3 (3.1%)		0 (0.0%)	3 (1.6%)
Miscellaneous	4 (5.1%)		3 (3.9%)		0 (0.0%)		4 (4.1%)		8 (9.0%)	12 (6.4%)
Post- bronchodilator test FEV1/FVC (%)	53.1±8.9	0.523	53.2±8.1	0.546	52.7±5.8	0.485	53.3±8.5	0.600	54.0±8.5	53.6±8.4
Post- bronchodilator test FEV1 % predicted	52.9±15.0	0.000*	49.5±13.5	0.000*	50.7±14.1	0.003*	52.5±14.0	0.000*	63.0±14.5	57.5±15.1
Positive bronchodilator test[&]	14 (17.9%)	0.562	15 (19.5%)	0.405	0 (0.0%)	0.207	18 (18.4%)	0.478	12 (13.5%)	30 (16.0%)
Airflow limitation severity (GOLD spirometric classification)		0.000*		0.000*		0.030*		0.000*		
I	3 (3.8%)		2 (2.6%)		1 (6.7%)		3 (3.1%)		15 (16.9%)	18 (9.6%)
II	41 (52.6%)		35 (45.5%)		8 (53.3%)		54 (55.1%)		59 (66.3%)	113 (60.4%)
III	30 (38.5%)		35 (45.5%)		5 (33.3%)		36 (36.7%)		15 (16.9%)	51 (27.3%)
IV	4 (5.1%)		5 (6.5%)		1 (6.7%)		5 (5.1%)		0 (0.0%)	5 (2.7%)
Co-morbidity										
Asthma	2 (2.6%)	1.000	2 (2.6%)	1.000	0 (0.0%)	1.000	2 (2.0%)	1.000	2 (2.2%)	4 (2.1%)
Ischemic heart disease	25 (32.1%)	0.117	22 (28.6%)	0.284	4 (26.7%)	0.517	29 (29.6%)	0.192	18 (20.2%)	47 (25.1%)
Congestive heart failure	26 (33.3%)	0.163	24 (31.2%)	0.276	4 (26.7%)	0.744	30 (30.6%)	0.275	20 (22.5%)	50 (26.7%)
Arrythmia	21 (26.9%)	0.627	21 (27.3%)	0.593	4 (26.7%)	0.744	25 (25.5%)	0.753	20 (22.5%)	45 (24.1%)
Chronic renal failure	29 (37.2%)	0.015*	26 (33.8%)	0.051	7 (46.7%)	0.041*	34 (34.7%)	0.026*	17 (19.1%)	51 (27.3%)
Malignancy	7 (9.0%)	1.000	5 (6.5%)	0.968	1 (6.7%)	1.000	8 (8.2%)	1.000	7 (7.9%)	15 (8.0%)
Length of hospital stay (days)	70.1±23.9	0.018*	64.1±25.1	0.323	64.0±31.1	0.626	66.8±25.3	0.088	59.7±31.2	63.5±28.4
Length of hospital stay before transfer to RCC (days)	24.7±10.2	0.843	25.0±11.4	0.982	29.3±13.2	0.210	25.0±11.2	0.963	25.1±11.7	25.0±11.4
Total length of use of IMV (days)	61.2±18.3	0.000*	57.5±20.4	0.000*	62.4±30.5	0.004*	57.2±19.8	0.000*	30.1±13.5	46.7±20.3
Length of use of IMV before transfer to RCC (days)	28.0±12.6	0.839	28.1±13.1	0.848	29.4±16.2	0.784	28.1±13.2	0.878	28.4±12.0	28.3±12.6
Modified GCS[#]	9.7±1.8	0.033*	9.8±1.8	0.088	9.4±1.3	0.047*	9.8±1.7	0.048*	10.2±1.4	10.0±1.6

APACHE II score	19.6±4.6	0.004*	19.4±4.4	0.008*	20.1±4.0	0.029*	19.5±4.6	0.005*	17.7±4.0	18.6±4.4
Laboratory findings										
WBC (10^9/L)	11.4±4.2	0.002*	11.9±4.7	0.000*	12.8±5.2	0.030*	11.6±4.5	0.000*	9.5±3.3	10.6±4.1
Blood eosinophil percentage (%)	2.4±3.0	0.752	2.0±2.9	0.211	1.5±1.4	0.142	2.3±3.0	0.510	2.5±2.8	2.4±2.9
Blood absolute eosinophil count (cells/μL)	248.0±293.8	0.645	199.5±239.7	0.490	165.9±139.7	0.402	229.2±284.1	0.968	227.6±277.4	228.4±280.2
Blood eosinophil percentage > 2%	35 (44.9%)	1.000	26 (33.8%)	0.244	5 (33.3%)	0.633	40 (40.8%)	0.789	39 (43.8%)	79 (42.2%)
Blood eosinophil percentage > 3%	20 (25.6%)	0.732	14 (18.2%)	0.140	1 (6.7%)	0.108	22 (22.4%)	0.373	26 (29.2%)	48 (25.7%)
Blood eosinophil percentage > 4%	12 (15.4%)	0.541	9 (11.7%)	0.202	1 (6.7%)	0.295	14 (14.3%)	0.377	18 (20.2%)	32 (17.1%)
Blood absolute eosinophil count > 150 cells/μL	38 (48.7%)	0.710	34 (44.2%)	0.339	9 (60.0%)	0.813	46 (46.9%)	0.512	47 (52.8%)	93 (49.7%)
Blood absolute eosinophil count > 200 cells/μL	35 (44.9%)	0.785	29 (37.7%)	0.723	7 (46.7%)	0.931	41 (41.8%)	1.000	37 (41.6%)	78 (41.7%)
Blood absolute eosinophil count > 300 cells/μL	21 (26.9%)	0.752	16 (20.8%)	0.804	1 (6.7%)	0.184	24 (24.5%)	1.000	21 (23.6%)	45 (24.1%)
Blood absolute eosinophil count > 400 cells/μL	17 (21.8%)	0.155	12 (15.6%)	0.708	1 (6.7%)	1.000	18 (18.4%)	0.352	11 (12.4%)	29 (15.5%)
Blood eosinophil percentage > 2% or absolute count > 200 cells/μL	39 (50.0%)	0.836	31 (40.3%)	0.459	7 (46.7%)	1.000	45 (45.9%)	0.978	42 (47.2%)	87 (46.5%)
Blood eosinophil percentage > 3% or absolute count > 300 cells/μL	24 (30.8%)	0.695	17 (22.1%)	0.102	2 (13.3%)	0.136	27 (27.6%)	0.359	31 (34.8%)	58 (31.0%)
Blood eosinophil percentage > 4% or absolute count > 400 cells/μL	19 (24.4%)	0.917	15 (19.5%)	0.779	2 (13.3%)	0.733	21 (21.4%)	1.000	20 (22.5%)	41 (21.9%)

Hemoglobin (g/dL)	9.8±1.4	0.060	9.9±1.4	0.082	9.4±0.9	0.029*	9.9±1.4	0.058	10.4±2.4	10.1±2.0
Albumin (g/dL)	2.9±1.0	0.932	2.7±1.0	0.043*	2.7±0.6	0.253	2.8±1.0	0.897	2.9±0.4	2.9±0.7
BUN (mg/dL)		0.003*		0.001*		0.009*		0.000*		
Available number	72 (92.3%)		70 (90.9%)		13 (86.7%)		90 (91.8%)		76 (85.4%)	166 (88.8%)
Mean±SD	48.0±41.5		51.6±42.8		79.5±54.9		51.1±42.3		31.5±20.0	42.1±35.3
Creatinine (mg/dL)	1.8±1.7	0.009*	1.6±1.6	0.025*	1.8±1.6	0.134	1.7±1.6	0.006*	1.2±1.0	1.5±1.4
pH	7.5±0.1	0.051	7.5±0.1	0.060	7.5±0.0	0.352	7.5±0.1	0.079	7.5±0.1	7.5±0.1
PaCO2 (mmHg)	39.7±8.8	0.029*	39.2±9.1	0.073	40.6±11.7	0.248	39.3±8.9	0.049*	36.8±7.7	38.1±8.4
PaO2/FiO2 ratio	292.6±154.6	0.695	286.2±150.2	0.512	290.0±142.4	0.786	291.0±150.9	0.623	302.4±165.5	296.4±157.7
CHOL (mg/dL)		0.009*		0.001*		0.011*		0.002*		
Available number	76 (97.4%)		76 (98.7%)		15 (100 %)		96 (98.0%)		88 (98.9%)	184 (98.4%)
Mean±SD	122.8±35.5		118.9±36.9		110.6±32.0		120.9±36.5		138.6±39.7	129.3±39.0
Prealbumin (g/L)		0.040*		0.011*		0.004*		0.018*		
Available number	77 (98.7%)		76 (98.7%)		15 (100 %)		97 (99.0%)		89 (100%)	186 (99.5%)
Mean±SD	0.1±0.1		0.1±0.1		0.1±0.1		0.1±0.1		0.2±0.1	0.2±0.1
COPD medications										
Use of systemic steroids	0 (0.0%)	NA	0 (0.0%)	NA	0 (0.0%)	NA	0 (0.0%)	NA	0 (0.0%)	0 (0.0%)
Use of short-acting bronchodilators	78 (100%)	NA	77 (100%)	NA	15 (100%)	NA	98 (100%)	NA	89 (100%)	187 (100%)
RSI※		0.852		0.998		0.435		0.923		
Available number	47 (60.3%)		41 (53.2%)		5 (33.3%)		55 (56.1%)		54 (60.7%)	109 (58.3%)
Mean±SD	158.4±129.9		153.6±133.3		200.4±134.3		156.0±126.3		153.6±126.7	154.8±125.9
Artificial airway		0.586		0.758		0.991		0.862		
Tracheostomy	54 (69.2%)		52 (67.5%)		9 (60.0%)		65 (66.3%)		57 (64.0%)	122 (65.2%)
Endotracheal tube	24 (30.8%)		25 (32.5%)		6 (40.0%)		33 (33.7%)		32 (36.0%)	64 (34.8%)
Ventilator settings										
Mode		1.000		0.793		0.225		0.679		
Volume control	55 (70.5%)		56 (72.7%)		13 (86.7%)		72 (73.5%)		62 (69.7%)	134 (71.7%)
Pressure control	23 (29.5%)		21 (27.3%)		2 (13.3%)		26 (26.5%)		27 (30.3%)	53 (28.3%)
Tidal volume (ml/kg)	9.7±0.7	0.755	9.7±0.7	0.413	9.9±0.5	0.599	9.7±0.7	0.675	9.8±0.6	9.8±0.7
Respiratory rate (breaths per minute)	17.3±1.0	0.141	17.2±1.0	0.470	17.3±1.0	0.323	17.3±1.0	0.150	17.1±1.0	17.2±1.0
Minute ventilation (liters per minute)		0.489		0.278		0.385		0.519		
Available number	38 (48.7%)		32 (41.6%)		4 (26.7%)		45 (45.9%)		45 (50.6%)	90 (48.1%)

Mean±SD	7.6±3.7		8.0±3.7		8.7±5.1		7.6±3.0		7.1±3.4	7.3±3.5
Use of NIPPV after successful liberation from IMV support during the RCC stay	28 (35.9%)	0.082	32 (41.6%)	0.013*	5 (33.3%)	0.348	37 (37.8%)	0.035*	20 (22.5%)	57 (30.5%)

*$p<0.05$ when compared to Individuals without any adverse outcomes.

[&]Positive bronchodilator test was defined as FEV 1 or FVC improvement from pre-dose value by ≧12% and ≧200 mL.

[#]Modified GCS, verbal score as one.

[※]RSI was defined as the ratio of respiratory frequency to tidal volume (breaths/minute/liter).

Abbreviations: see Supplementary Table S1.
